# Immune checkpoint inhibitor-associated Vogt-Koyanagi-Harada-like syndrome: A descriptive systematic review

**DOI:** 10.1186/s12348-025-00484-8

**Published:** 2025-05-12

**Authors:** Huixin Anna Zhang, Amelia T. Yuan, Noémie Chiasson, Kevin Y. Wu, Ananda Kalevar

**Affiliations:** 1 Department of Ophthalmology and Vision Sciences, University of Toronto, Toronto, ON Canada; 2 Faculty of Medicine, Université Laval, Quebec, QC Canada; 3 Schulich School of Medicine and Dentistry, University of Western Ontario, London, ON Canada; 4 Department of Medicine, Université de Sherbrooke, Sherbrooke, QC Canada; 5 Department of Surgery, Division of Ophthalmology, University of Sherbrooke, Sherbrooke, QC Canada; 6 Axe Visuel, 1290 Rue Belvédère S, Sherbrooke, QC J1H 4C7 Canada

**Keywords:** Immune checkpoint inhibitor, Vogt-Koyanagi-Harada-like uveitis, Panuveitis, Drug-induced, Immune related adverse event

## Abstract

**Topic:**

Vogt-Koyanagi-Harada (VKH)-like uveitis is uniquely reported with immune checkpoint inhibitors (ICI) and BRAF/MEK inhibitors. This article aims to provide a comprehensive portrait of the comorbidities, ocular presentations, treatments, and visual outcomes of patients with VKH-like uveitis following ICI therapy.

**Clinical relevance:**

ICIs are increasingly used in cancer therapy, but poorly understood ocular immune-related adverse events (irAEs) can lead to suspension of treatment and be vision-threatening.

**Methods:**

We conducted a systematic review (PROSPERO #CRD42024558269) according to PRISMA guidelines. MEDLINE, Embase, CENTRAL, and Web of Science were searched for English articles published up to June 28, 2024. All study designs reporting on incident VKH-like uveitis following ICI were included. Risk of Bias was assessed using a tool modified from Murad et al. (2018).

**Results:**

Of 865 articles, we included 42 articles (4 observational studies, 28 case reports, 6 case series, 3 letters, and 1 editorial) from 12 countries, comprising 52 patients. The mean age was 60.0 ± 11.9 years, and 32 (61.5%) were females. Thirty-six (69.2%) had melanoma, and most were undergoing treatment with a PD-1 inhibitor alone (*n* = 33, 63.5%) or in combination with a CTLA-4 inhibitor (*n* = 10, 19.2%). The mean duration of ICI treatment before VKH-like uveitis symptoms was 22.2 ± 29.6 weeks, and the mean duration of ocular symptoms was 16.7 ± 18.6 weeks, with wide variation. Overall, 43 patients (73.1%) had imaging or exams suggesting bilateral involvement and 21 cases (40.4%) suggesting panuveitis. Only 31 cases (59.6%) met the acute initial-onset uveitis criteria, and 15 (28.8%) met the chronic phase criteria. Most (*n* = 47, 90.4%) required systemic or intravitreal steroids, termination of ICI (*n* = 31, 59.6%), and experienced full resolution or remission of visual symptoms (*n* = 43, 82.7%). Most articles (*n* = 40, 95.2%) were judged to be at medium risk of bias.

**Conclusion:**

This descriptive systematic review consisted mostly of case reports, but it confirmed that a high proportion of VKH-like uveitis occur with PD-1 inhibitors and melanoma patients. VKH-like uveitis can lead to suspension of treatment. Further collaboration between oncologists and ophthalmologists is needed in the continuum of cancer care.

**Supplementary Information:**

The online version contains supplementary material available at 10.1186/s12348-025-00484-8.

## Introduction

Although ocular inflammation induced by immune checkpoint inhibitors (ICI) is rare, occurring in less than 1% of cases, they can leave debilitating marks on vision [1]. Uveitis represents the most common irAEs in ocular condition [1]. Among manifestations of ICI-induced uveitis, posterior or panuveitis with Vogt- Koyanagi-Harada (VKH) disease-like characteristics have been reported. This is an entity uniquely associated with BRAF/MEK and ICI administration, however little is known about their risk factors, treatments, and outcomes [2, 3]. 

Clinical manifestations of VKH disease differ, but the diagnostic criteria for the acute initial-onset and the late-phase of VKH disease as defined in Yang et al. (2018) and Herbort et al. (2022) are detailed in Table 1 [4, 5]. There are four stages of VKH disease. The prodromal stage occurs first and is accompanied by flu-like symptoms, headache, meningismus, or back stiffness [6, 7]. This is followed by the acute initial-onset uveitis associated with VKH disease. This stage is characterized by bilateral granulomatous choroiditis with secondary exudative retinal detachments associated with optic nerve head inflammation. Choroidal involvement can be assessed by indocyanine green angiography (ICGA) or OCT Enhanced Depth Imaging (EDI-OCT) [5]. Next, the late phase with cutaneous and ocular depigmentation may occur over a period of weeks to months. Finally, the recurrent stage follows wherein inflammation recur and complications such as glaucoma, cataract, and choroidal neovascularization may develop [8]. The prognosis of VKH disease is usually positive with early treatment, with up to 60–70% achieving a visual acuity of 20/40 [9–11]. While the usual treatment regimen involves concentrated systemic corticosteroids with slow tapering, many studies have shown that introducing systemic immunosuppressants early in the disease course (between 2 and 4 weeks) is essential to managing initial-onset VKH and improving outcomes [12, 13]. 

**Table 1:** Diagnostic criteria of VKH disease, adapted from Yang et al. (2018)

Diagnostic Criteria for Vogt-Koyanagi-Harada (VKH) Disease				
A. No history of penetrating ocular trauma or intraocular surgery preceding the initial onset of uveitis				
B. Bilateral ocular involvement				
C. No evidence of infectious uveitis or accompanying systemic rheumatic diseases or evidence suggestive of other ocular disease entities				
D. Early-phase VKH disease:	1. Signs of diffuse choroiditis and exudative retinal detachment			
	2. Serous retinal detachment on OCT or B-scan ultrasonography			
	3. Choroidal thickening on EDI-OCT or B-scan			
	4. Early punctate staining and late subretinal dye pooling on FFA			
	5. Hyperfluorescence of the optic disc on FFA			
	Definite diagnosis:	Variant 1: In patients presenting with A + B + C + D(1)		
		Variant 2: In patients without clinically visible exudative retinal detachment, ie, A + B + C + D(2) + D(3) or A + B + C + D(4)		
		Variant 3: In patients already treated with systemic corticosteroids or combined with other immunosuppressive agents, a history of typical appearances of variant 1 or 2, and A + B + C + D(5)		
E. Late-phase VKH disease				
	1. Signs of definite sunset glow fundus or retinal pigment epithelium clumping/migration			
	2. Signs of bilateral recurrent granulomatous anterior uveitis			
	3. Signs of Dalen-Fuchs nodules or multifocal chorioretinal atrophy			
	4. Window defects/moth-eaten fluorescence on FFA			
	5. Previous history of characteristic findings corresponding to diagnosis of early-phase VKH disease			
	Definite diagnosis:	Variant 1: In patients presenting with A + B + C + E(1) + E(2)		
		Variant 2: In patients without sunset glow fundus or visible pigment alternations due to early and appropriate treatment, ie, A + B + C + E(2) + E(3) or A + B + C + E(2) + E(4)		
		Variant 3: In patients with significant media opacity, ie, A + B + C + E(2) + E(5)		

Abbreviations: EDI, enhanced depth imaging; FFA, fluorescence fundus angiography; OCT, optical coherence tomography

In a FDA pharmacovigilance study of 41,674 cancer patients receiving ICI, the incidence of VKH-like uveitis was described to be 8.3% among all uveitis cases, of which 68.8% were receiving treatment for melanoma [14]. Another study found that half of VKH-like uveitis were undergoing treatment for melanoma [15]. VKH-like uveitis is uniquely associated with cutaneous or choroidal melanoma, leading to the hypothesis that its pathogenesis involves cross-reactivity between pigment and cancerous tissues, leading to a proinflammatory and autoimmune response [2, 9]. Consequently, its occurrence may be a positive prognosis factor in ICI response, but this has never been shown. Interestingly, in a WHO pharmacovigilance report, 8 VKH-like uveitis were reported, and only one experienced significant visual loss [3]. Despite this, most cases of drug-related side effects are not reported to pharmacovigilance databases, especially if they are not severe: in a French tertiary oncology center, only 30% of the severe adverse events secondary to ICI were reported [16]. More importantly, the detailed clinical presentation, treatment, and prognosis of these VKH-like uveitis cases have never been summarized.

While VKH disease is an autoimmune condition directed against melanocytes, VKH-like disease is reported to occur as an adverse event following exposure to ICI therapy. In this systematic review, we aim to summarize the clinical presentations, treatments, and outcomes of cases reported in the literature as “VKH-like uveitis” associated with prior or concurrent use of ICI. This is the first review to comprehensively describe the presentation and management of all VKH-like uveitis reported to date in the context of ICI therapy.

## Methods

This systematic review was conducted according to the “Preferred Reporting Items for Systematic Reviews and Meta-analyses” (PRISMA) guidelines [17]. The study was prospectively registered on PROSPERO on July 5, 2024 (registration number CRD42024558269).

### Data sources

A comprehensive database search was performed using a Boolean approach with predefined search terms in Medline, Embase, Web of Science, and Cochrane Library on June 28, 2024. The search strategy was developed with the help of a librarian and included in Figure S1. In addition, reference lists were reviewed to include relevant reports.

### Inclusion and exclusion criteria

To be included in this review, articles had to describe patients over 18 years old diagnosed by authors as having “VKH-like uveitis” as the most probable diagnosis during or after ICI use. Patients with a history of VKH-like uveitis prior to ICI use, diagnosis of VKH-like uveitis unrelated to ICI, and non-ICI use were excluded. We also excluded non-English articles, and articles where the full text was not found or the abstract did not report the relevant outcomes. All study designs, including case reports, case series, correspondences, commentaries, and letters to the editor were included in this review.

### Study selection

Articles were screened based on titles and abstracts by two independent reviewers (AZ, NC). The same two reviewers evaluated full-text studies based on the inclusion and exclusion criteria above. Any disagreement was resolved by consensus between the two reviewers, and a third reviewer (KYW) was consulted if consensus could not be reached.

### Data extraction

Two groups of independent reviewers (AZ, AY, NC) extracted data from the included studies on a pre-determined Microsoft Excel table. The table was piloted after the extraction of three initial articles. Data on study characteristics (publication year, country, study design) and patient characteristics (age, sex, ethnicity, medical history, etc.) were collected. Data on ICI use (cancer diagnosis/stage, medication type, course, dosage), VKH-like uveitis (clinical signs & symptoms, diagnostic tests, course of illness), and outcomes (treatment regimen, treatment duration, ocular outcome, general outcome) were recorded as primary outcomes. Missing information were noted as not reported (NR).

### Quality assessment and risk of bias

Qualified studies were independently assessed by two groups of independent reviewers (AZ, AY, NC) for the quality of evidence. There was a total of 9 items to be assessed. The risk of bias and certainty of evidence were assessed by using a quality assessment scale and GRADE approach adapted from Murad et al. (2018) [18, 19]. Any disagreements were resolved by consensus between the two reviewers.

### Data synthesis and statistical analysis

All included data were synthesized. Missing data were reported, and their sources cited in-text for main outcome items. Tabulation was completed using filters and formulas on Microsoft Excel. Descriptive statistics was used to report the clinical and demographic data of the current study. Means and standard deviations were used for continuous variables, whereas percentages were used for categorical variables. One-way ANOVA to compare sub-groups was performed using GraphPad Prism version 10.4.0 for Mac, GraphPad Software, Boston, Massachusetts USA, www.graphpad.com. Sensitivity analyses were not carried out due to the nature of included articles.

## Results

**Fig. 1:**
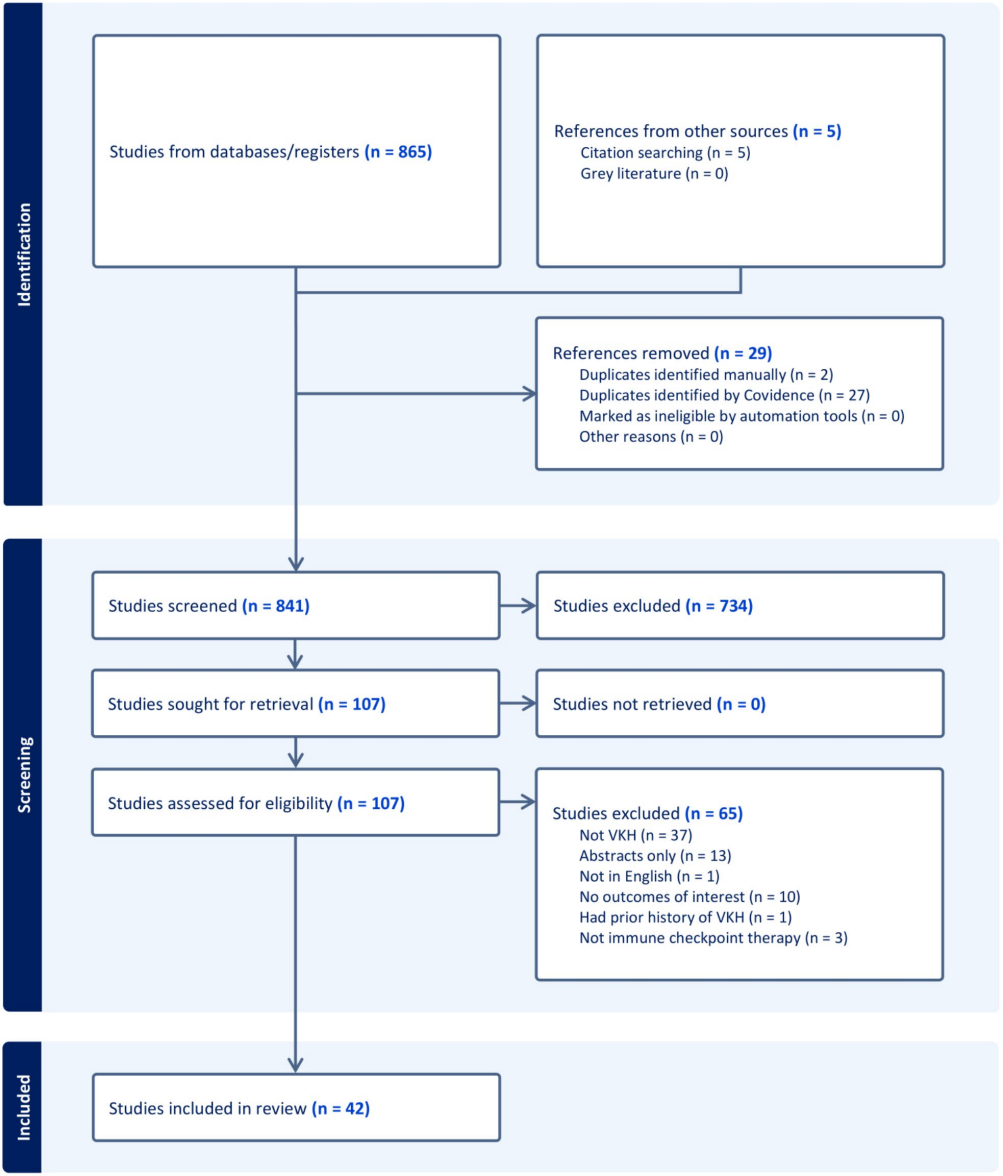
Flow diagram of literature search and selection of studies according to PRISMA

A total of 865 articles were identified through database searches. After eliminating duplicate studies, 836 titles and abstracts, and 102 full-text articles were screened. A total of 42 studies with 52 patients were included in this manuscript (Fig. 1) [20–62]. Studies reporting on VKH-like panuveitis symptoms not attributed to immune checkpoint inhibitors or reactions or VKH-like uveitis triggered by other medications were excluded [45, 63–65]. 

Of the studies included, 29 (69.0%) were case reports [20, 22, 25, 28, 30, 31, 33–36, 38–44, 46–48, 50–57, 62]. Six (14.3%) were case series [23, 24, 29, 30, 37, 49]. Four (9.5%) were observational studies reporting individual patient characteristics [58–61]. These reports were spread across many different institutions around the world, with the majority originating from Japan (*n* = 15, 35.7%) and the United States (*n* = 14, 33.3%). The characteristics of the remaining reports are found in Table 2.

**Table 2:** Journal information

Variables	Frequency (%)
Country	
Japan, n (%)	15 (35.7)
USA, n (%)	14 (33.3)
Germany, n (%)	3 (7.1)
France, n (%)	2 (4.8)
Belgium, n (%)	1 (2.4)
China, n (%)	1 (2.4)
Ireland, n (%)	1 (2.4)
Portugal, n (%)	1 (2.4)
South Korea, n (%)	1 (2.4)
Spain, n (%)	1 (2.4)
Switzerland, n (%)	1 (2.4)
UK, n (%)	1 (2.4)
Study design	
Case report, n (%)	29 (69.0)
Case series, n (%)	6 (14.3)
Editorial, n (%)	1 (2.4)
Letter	3 (7.1)
Observational study, n (%)	4 (9.5)

### Demographic characteristics

The demographics of the patients reported in the studies are summarized in Table 3. The mean age was 60.0 ± 11.9 years old, and the majority of patients were female (61.5%). The ethnicities of the patients were unreported for 51.9% of the studies included. Of those reporting ethnicities, Caucasian patients (*n* = 13, 25.0%) made up the majority [20, 22, 24, 26, 33, 36, 40, 58, 59], followed by Asian (*n* = 10, 19.2%) [21, 30, 51, 60, 61] and Hispanic patients (*n* = 2, 3.8%) [44, 47]. The type of cancer for which ICI was indicated was melanoma [20–33, 36, 37, 40, 42, 46–49, 55–62] in over two thirds of cases (*n* = 36, 69.2%), followed by renal cell carcinoma (*n* = 6, 11.5%) [39, 44, 52, 59, 60] and lung cancers (*n* = 6, 11.5%) – including non-small cell lung cancer [38, 50, 51], small cell lung cancer [59, 60], and lung adenocarcinoma [53]. Other types of cancer included ovarian [34], gastric [43, 60], hypopharyngeal [35], and bladder urothelial carcinoma [54]. 

**Table 3:** Socio-demographic summary of cohort

Variables	Frequency (%)
Age (Years)	
mean ± SD	60.0 ± 11.9
Sex	
Male, n (%)	20 (38.5)
Female, n (%)	32 (60.0)
Reported Ethnicity	
Caucasian (Non-Hispanic white), n (%)	13 (25.0)
Asian, n (%)	10 (19.2)
Hispanic, n (%)	2 (3.84)
Unknown, n (%)	27 (51.9)
Cancer types	
Melanoma	36 (69.2)
Renal cell carcinoma	6 (11.5)
Lung cancer	6 (11.5)
Other cancer	5 (9.6)
Duration of treatment before symptoms (weeks)	
mean ± SD	22.2 ± 29.6
Duration of VKH symptoms (weeks)	
mean ± SD	16.7 ± 18.6
Duration of follow up (months)	
mean ± SD	11.7 ± 9.7

### Relevant medical & ocular history

Of 52 patients included, additional past medical history was reported in 15 patients (28.8%). Among these, one patient had iron deficiency anemia [44], one was a smoker [51], and one had type II diabetes mellitus and hypercholesteremia [47], one had hypothyroidism and hypercholesteremia [56], one had congenital hearing loss [52]. The other ten patients reported no prior autoimmune diseases, infections, or other relevant past medical history [20, 31, 33, 34, 36, 40, 43, 59]. Past ocular history was reported in 18 patients (34.6%). Among these, cataract surgery was reported in 3 (5.7%), [41, 48, 52] prior primary VKH disease without recurrence was reported in two (3.8%), [60] Ahmed valve implantation for steroid-induced secondary glaucoma was reported in one [34], vitrectomy for retinal detachment was reported in one [48], and no relevant ocular history was reported in 11 patients [20, 25, 28, 31, 34–36, 40, 46, 54, 56]. Of 53 cases, none reported a family history of autoimmune diseases, uveitis, or VKH disease.

### ICI characteristics

ICI types that preceded the VKH symptoms are shown in Table 3. PD-1 inhibitors were the most common and accounted for almost two thirds of cases (*n* = 33) [21, 22, 24, 26–28, 30, 31, 33–35, 38–41, 43, 44, 46–48, 51–54, 58–61]. Specifically, 19 patients (36.5%) used nivolumab [21, 30, 31, 34, 35, 39–41, 43, 44, 46, 47, 52, 53, 59, 60], 12 (23.1%) used pembrolizumab [22, 24, 26–28, 38, 48, 51, 58, 60, 61], one (1.9%) used cemiplimab [33], and one (1.9%) used toripalimab [54]. This was followed by anti-CTLA-4 and anti-PD-1 combination therapy (*n* = 10, 19.2%) [23, 32, 36, 37, 42, 49, 58–60, 62]. All combination therapy patients used ipilimumab and nivolumab. CTLA-4 inhibitor alone (*n* = 7, 13.5%) [25, 29, 55–58] and PD-L1 inhibitor alone (*n* = 2, 3.8%) [50, 60] accounted for only 17.3% of cases. The mean duration of cancer treatment prior to VKH symptoms was 22.2 ± 29.6 weeks. The detailed demographics of the patients included are presented in Tables 4 and 5, with the specific checkpoint inhibitor drugs used listed.

**Table 4:** Summary of ICI therapy information

Variables	Frequency (%)
Type of ICI	
PD-1 inhibitor, n (%)	33 (63.5)
Combined CTLA-4 & PD-1 inhibitors, (%)	10 (19.2)
CTLA-4 Inhibitor, n (%)	7 (13.5)
PD-L1 inhibitor, n (%)	2 (3.8)
Names of ICI	
Nivolumab, n (%)	19 (36.5)
Pembrolizumab, n (%)	12 (23.1)
Ipilimumab + Nivolumab combo, n (%)	10 (19.2)
Ipilimumab, n (%)	7 (13.5)
Atezolizumab, n (%)	1 (1.9)
Cemiplimab, n (%)	1 (1.9)
Durvalumab, n (%)	1 (1.9)
Toripalimab, n (%)	1 (1.9)

**Table 5:** Detailed demographics, ICI treatment, clinical findings, and outcomes of cases reported as VKH-like uveitis

Author and Year	Age Sex	Cancer Type	ICI Name	Ophthalmic Presentation	Slit Lamp Findings	Fundoscopy Findings	Imaging Findings	Acute-initial onset VKH?	Chronic phase?	Extraocular Manifestations	Treatment	Time to Uveitis Treatment (weeks)	Neoplastic Outcome	Visual Outcome
Arai 2017 [21]	55 M	Melanoma	Nivolumab	Blurry vision	OU: Mild inflammatory reaction with fibrin formation, posterior synechia in AC	NR	NR	No	No	Vitiligo;	Topical steroids and mydriatics	NR	NR	Resolution
Bricout 2017 [22]	59 M	Melanoma	Pembrolizumab	Vision loss, eye redness	OU: AC cell, nongranulomatous anterior uveitis	OU: Exudative RD with 360-degree choroidal detachment.	OCT: Choroidal folds, retinal nerve fiber layer was 111 um OD, 110 um OS. B-scan: choroidal detachment with no sign of posterior scleritis. Little subretinal fluid in both eyes. FFA: OU papillary edema, disc leak.	Variant 1 Variant 3	No	Poliosis; Vitiligo; CSF pleocytosis;	Topical dexamethasone and subconjunctival betamethasone injection	NR	Partial remission	NR
Chaudot 2022 [23]	59 F	Melanoma	Ipilimumab and Nivolumab	NR	OU: Anterior uveitis	OU: Multiple white spots on posterior pole.	NR	No	No	Vitiligo; CSF pleocytosis;	IV, PO, topical steroids	NR	Partial remission	Resolution
Conrady 2017 [24]	57 M	Melanoma	Pembrolizumab	Vision loss	Unremarkable	OU: Optic disc hyperemia, choroidal folds, subretinal fluid.	OCT: Significant subretinal fluid.	Variant 1	No	NR	PO steroids	2	Complete remission	Resolution
Conrady 2017 [24]	78 F	Melanoma	Pembrolizumab	Vision loss, photophobia, panuveitis	OU: Ciliary body detachment, panuveitis	OU: Panuveitis. RD.	OCT: Large choroidal fluid, ciliary body detachments.	Variant 1	Variant 3	Hearing loss;	IV, PO, topical steroids	NR	NR	NR
Crosson 2015 [25]	54 F	Melanoma	Ipilimumab	Blurry vision	Unremarkable	OU: Diffuse patches of choroidal hypopigmentation, RPE changes in circular configurations. Sunset flow fundus with macular RPE mottling.	FFA: Early hypofluorescence and late hyperfluorescence in areas of RPE changes, regressed multifocal exudative RDs.	Variant 2	No	Poliosis; Vitiligo; Tinnitus; Headache;	Surveillance	NR	NR	NR
de Vries 2022 [26]	25 F	Melanoma	Pembrolizumab	Vision loss, eye redness	OU: AC cells, mild vitreous cells	OU: Disc swelling, serous RD. peripapillary atrophy following steroids.	ICGA: Patchy choroidal filling. FFA: Patchy choroidal filling.	Variant 1 Variant 2 Variant 3	No	Headache;	PO and topical prednisone		Partial remission	Resolution
Dolaghan 2019 [27]	77 F	Melanoma	Pembrolizumab	Blurry vision	NR	OU: Exudative RDs, worse in OS.	OCT: Intra-retinal fluid.	Variant 1	No	Poliosis; Hearing loss;	PO steroids and topical prednisolone acetate	NR	Complete remission	Recurrence
Enomoto 2020 [28]	68 F	Melanoma	Pembrolizumab	Vision loss	OU: Shallow AC	OU: Choroidal folds. OD: Serous RD.	AS-OCT: Ciliary body edema, ciliary body detachment OD; thickened choroid and choroidal folds; serous/fluid RD OD extensive. ICGA: Hypofluorescent dark area within vascular arcade OD. FFA: Hyperfluorescence OD, leak of fluorescein on optic discs OU.	Variant 1 Variant 2 Variant 3	Variant 2 Variant 3	Tinnitus; Meningismus;	PO prednisone and topical betamethasone	NR	NR	Partial resolution
Fierz 2016 [29]	43 M	Melanoma	Ipilimumab	Blurry vision	OU: Bilateral panuveitis, 2 + AC cells and 4 + AC flare with fibrin	OU: Disc swelling, white choroidal lesions. OD: Peripapillary choroidal folds.	ICGA: Hypofluorescent spots appeared early and remained in late frames. Peripapillary choroidal folds OD.	Variant 1	Variant 2 Variant 3	NR	Topical prednisolone and PO prednisone	NR	Complete remission	Resolution
Fujimura 2018 [30]	73 M	Melanoma	Nivolumab, dabrafenib and trametinib	Visual impairment	NR	OU: Entire circumference serous RD.	OCT: OU diffuse thickening of choroid, RD on MRI.	Variant 1 Variant 2	No	Vitiligo; Hearing loss;	PO prednisone and IV methylprednisolone	NR	Partial remission	Partial resolution
Fujimura 2018 [30]	35 F	Melanoma	Nivolumab, dabrafenib and trametinib	Visual impairment	NR	OU: Entire circumference serous RD.	NR	Variant 1	No	Hearing loss; Headache;	IV methylprednisolone	NR	Complete remission	Partial resolution
Gambichler 2020 [31]	63 F	Melanoma	Nivolumab	Blurry vision	OU: Disc edema, anterior/intermedia uveitis	OU: Disc swelling	NR	No	No	Vertigo; Hearing loss;	IV methylprednisolone, then PO prednisolone	NR	NR	Partial resolution
Godse 2021 [32]	57 F	Melanoma	Nivolumab and ipilimumab	Blurry vision, light sensitivity	OU: Mutton-fat KP (granulomatous uveitis)	NR	NR	No	No	Alopecia; Poliosis; Vertigo; Hearing loss;	PO prednisone and MMF	NR	Complete remission	Resolution
Huang 2023 [33]	53 F	Melanoma	Cemiplimab	Blurry vision, ocular pain	OU: 1 + AC and vitreous cells	OU: Disc edema and choroidal folds in macula. Choroidal depigmentation. Sunset glow fundus centrally with peripheral RPE clumping and migration.	FFA: OU trace optic nerve staining. OS window defect in the venous phases likely due to RPE atrophy.	No	No	Alopecia; Vitiligo;	PO prednisone, azathioprine, MMF, ADA, sub-tenon triamcinolone	NR	Partial remission	Partial resolution
Hwang 2022 [34]	61 F	Ovarian	Nivolumab	Blurry vision	Unremarkable	OU: Optic disc edema with choroidal swelling on the posterior pole.	OCT: Massive subretinal fluid accumulation, choroidal thickening. FFA: Multiple leakage points of fluorescein dye with optic disc staining in late phase.	Variant 1 Variant 2 Variant 3	No	Headache;	IV, then PO prednisolone pulse therapy	2	Progression	Recurrence
Kikuchi 2020 [35]	63 M	Hypopharyngeal	Nivolumab	Blurry vision	OU: Anterior uveitis, granulomatous mutton-fat KP, AC cells	OU: Disc swelling and serous RD. Sunset glow fundi at last visit.	OCT: Subretinal fluid, optic disc swelling. ICGA: Hypofluorescent dark dots in the intermediate phase and incompetency of choroidal circulation. FFA: Small leakages of fundi and optic disc.	Variant 1 Variant 2 Variant 3	Variant 1 Variant 2 Variant 3	NR	Sub-tenon triamcinolone, IV then PO methylprednisolone pulse therapy	1	Complete remission	Resolution
Kilani 2023 [36]	52 F	Melanoma	Nivolumab and ipilimumab	Blurry vision, visual impairment	Unremarkable	OU: Mild narrowing of the retinal arterial vessels and scarce vitrous cells. At follow-up, small patches of hypoautofluorescence surrounded by a ring of hyperautofluorescence.	OCT: Pachychoroidal changes, discrete choroi- dal folds and subretinal fluid OU. ICGA & FFA: Choroidal congestion late phase and discrete extravascular hyperfluorescence without retinal vasculitis or loss of choroidal vascular details.	Variant 1 Variant 2	No	NR	PO, IV methylprednisolone	3	Death	Resolution
Kim 2019 [37]	43 F	Melanoma	Nivolumab and ipilimumab	General VKH symptoms	NR	NR	NR	No	No	Vitiligo;	Systemic corticosteroid	NR	Partial remission	Partial resolution
Kurono 2020 [38]	71 M	NSCLC	Pembrolizumab	Visual impairment	OU: Anterior granulomatous uveitis with mutton-fat KP	OU: Posterior synechia and RD.	OCT: Posterior synechia and RD. FFA: Disseminated spotted choroidal hyperfluorescence.	Variant 1 Variant 2	Variant 2 Variant 3	Alopecia; Hearing loss; Headache; CSF pleocytosis;	PO prednisone, IV pulse methyprednisolone	NR	NR	Resolution
Lima 2024 [39]	55 F	Clear Cell RCC	Nivolumab	Vision loss	NR	OU: Thickening of choroid.	OCT: Thickening of choroid.	No	No	NR	Intravitreal corticosteroids	NR	Progression	Resolution
Madoe 2023 [40]	49 M	Melanoma	Nivolumab	Blurry vision	OU: Mild anterior uveitis	OU: Optic disc swelling, bilateral serous RD (large central OD, small multifocal OS peripapillary and perimacular region).	OCT: Subretinal fluid, undulating RPE, multifocal choroidal thickening, optic disc swelling OU. FFA: Disc edema, leakage at border of subretinal fluid OU.	Variant 1 Variant 2 Variant 3	Variant 3	NR	Topical dexamethasone, PO methylprednisolone	NR	Partial remission	Partial resolution
Matsuo 2017 [41]	60 F	Melanoma	Nivolumab	Xanthopsia	OU: Unremarkable initially but 2 + mutton-fat KP. Whitish iris nodules during recurrence	OU: Elevations of retina bilaterally. Red depigmented fundi during recurrence.	OCT: Wavy RPE line with multifocal choroidal thickening OU; subretinal fluid OD.	No	No	Poliosis;	Topical betamethasone, PO prednisone	1	Progression	Partial resolution
Minami 2021 [42]	73 M	Melanoma	Nivolumab and ipilimumab	Visual impairment and floaters	OU: Moderate granulomatous anterior uveitis.	OU: Hyperemia of optic disc, multiple serous RDs. Mild sunset glow fundus at last visit.	OCT: Undulation of RPE, serous RDs with septum, increase in choroidal thickness. ICGA: Multiple hypofluorescent dark spots during late phase. FFA: Late leakage and pooling, optic disc hyperfluorescence.	Variant 1 Variant 2 Variant 3	Variant 1 Variant 3	NR	Sub-tenon triamcinolone, topical betamethasone	2	Complete remission	Resolution
Monferrer-Adsuara 2021 [20]	64 F	Melanoma	Ipilimumab	Blurred vision, eye redness	OU: 2 + cells, diffuse granulomatous KP, posterior synechia.	OU: Intense vitritis OS: Disc edema	FFA: OU papillitis (late phase hyperfluorescence of the optic disc).	Variant 3	No	Poliosis; Vitiligo; Vertigo; Hearing loss; Headache;	Topical prednisolone, and PO prednisolone	NR	Complete remission	Resolution
Nagai 2023 [43]	72 M	Gastric Cancer	Nivolumab	Blurry vision	OU: AC cells, KP.	OU: Serous RD, wavy RPE, choroidal thickening.	OCT: Serous RDs, wavy RPE choroidal thickening. ICGA: Hypofluorescent dark spots during late phase. FFA: Leakage in small areas, pooling on posterior pole, hyperfluorescent optic discs.	Variant 1 Variant 2 Variant 3	Variant 1 Variant 2 Variant 3	Hearing loss; CSF pleocytosis;	Topical betamethasone, PO prednisone, sub-tenon triamcinolone, tropicamide, phenylephrine	2	Progression	Partial resolution
Ng 2023 [44]	49 F	Clear Cell RCC	Nivolumab	Temporal photopsia	Unremarkable	OU: Sunset glow fundus & diffuse choroidal hypopigmentation at 7mo FU. OD: Elevated lesion temporal to the macula with subretinal fluid OD.	OCT: choroidal folds OU, RD OD. thickened underlying choroid. ICGA: Hypofluorescent dark spots during late phase OD. At recurrence, mottled hypofluorescence and punctate foci of hyperfluorescence. FFA: Pooling of dye at bacillary layer. At recurrence, hyper-autofluorescence at the area of the lesion and subretinal fluid.	Variant 1 Variant 2	Variant 1 Variant 2 Variant 3	NR	PO prednisone	11	NR	Resolution
Noble 2020 [58]	62 F	Choroidal melanoma	Ipilimumab and Nivolumab	Central scotoma	NR	OS: Multiple pockets of exudative RD.	OCT: OS pockets of central subretinal fluid with multiple areas of localized retinal elevation.	No	No	Poliosis; Vitiligo;	PO corticosteroids	NR	NR	Partial resolution
Noble 2020 [58]	63 M	Melanoma	Pembrolizumab	Visual impairment, light sensitivity, eye redness	NR	OS: VKH-like reaction	NR	No	No	NR	Topical corticosteroid	NR	NR	Resolution
Noble 2020 [58]	30 F	Melanoma	Ipilimumab	Blurry vision and infection	OU: Recurrent anterior uveitis.	OU: VKH-like reaction	NR	No	No	NR	Topical corticosteroids and PO steroids	NR	NR	Resolution
Obata 2019 [46]	63 F	Melanoma	Nivolumab	Vision loss	OU: Granulomatous KP and cells.	OU: Mild vitreous opacity in inferior quadrant. At 4 months, fundi appeared red and depigmented. OS: Multiple sites of serous RD and wavy RPE.	OCT: Multiple sites of serous RD OS and wavy RPE OU. Choroid thickness 369.3µm in the right eye and 663.3µm in the left eye. FFA: Pinpoint-sized areas of leakage and active leakage from disc OD. Choroidal hyperfluorescence due to choroidal vascular leakage and hypofluorescent dark spots.	Variant 1 Variant 2 Variant 3	Variant 1 Variant 2 Variant 3	Poliosis; Headache;	Topical betamethasone, tropicamide, phenylephrine	1.5	Progression	Partial resolution
Pole 2023 [47]	63 F	Melanoma	Nivolumab	Vision loss	OD: 2 + cell, 1 + vitreous cell.	OU: RPE changes, focal hypopigmented lesions, microaneurysms, dot-blot hemorrhages, normal optic nerves.	OCT: Normal lining of the RPE.	No	No	Poliosis;	IV corticosteroids	24	NR	Partial resolution
Qian 2024 [59]	67 F	RCC	Nivolumab	NR	OU: VKH-like panuveitis.	OU: Intraretinal and subretinal fluid, papillitis. OD: Choroidal neovascularization.	NR	Variant 1	No	NR	Topical steroid, PO steroid, infliximab	NR	NR	Resolution
Qian 2024 [59]	81 F	SCLC	Nivolumab	NR	OU: Anterior uveitis with posterior synechiae.	OU: VKH-like panuveitis with serous RD.	NR	Variant 1	No	NR	Topical corticosteroid, PO steroids	NR	NR	Resolution
Qian 2024 [59]	66 F	Melanoma	Ipilimumab and Nivolumab	Eye redness and blurry vision	OU: Anterior uveitis.	NR	NR	No	No	Poliosis; Vitiligo;	Topical corticosteroid, PO steroids		Complete remission	Resolution
Rapisuwon 2019 [62]	60 F	Uveal melanoma	Ipilimumab and Nivolumab	NR	NR	OS: Central serous retinopathy, RD.	OCT: Central serous retinopathy/subretinal fluid.		No	Vitiligo; Tinnitus;	NR	NR	NR	Resolution
Reid 2019 [48]	73 M	Melanoma	Pembrolizumab	Visual impairment	OU: Non-granulomatous inflammation AC and anterior vitreous cells. OS: Posterior synechiae	OU: Choroidal effusions, ciliary body detachments. Disc swelling.	OCT: Choroidal thickening. No RDs. ICGA: No leakage or hypoperfusion.	No	No	Poliosis; Hearing loss;	IV methylprednisolone, peribulbar triamcinolone acetonide, OVD injections in the AC	1	Complete remission	Resolution
Sada 2023 [61]	57 M	Melanoma	Pembrolizumab	NR	OU: AC cells > 2+. Anterior/intermediate uveitis.	OU: Multifocal serous RD and choroidal folds.	OCT: Thickened choroid and choroidal folds. ICGA: Multiple hypofluorescence dark spots. FFA: Spotted hyperfluorescence during the early phase and leakage of fluorescein in the late phase.	Variant 1 Variant 2	Variant 3	Poliosis; Vitiligo;	Topical steroids, systemic steroids, methotrexate	NR	Complete remission	Resolution
Sturmer 2021 [49]	55 F	Melanoma	Ipilimumab and Nivolumab	Blurred vision	NR	NR	NR	No	No	Vertigo; Hearing loss;	Topical prednisolone acetate, PO prednisolone	0.3	Partial remission	Resolution
Suwa 2021 [50]	76 F	NSCLC	Atezolizumab	Visual impairment	OU: Fibrin deposits on anterior surface of lens, inflammation in AC.	OU: Swelling of optic disc.	OCT: Multiple serous RDs, wavy RPE, choroidal thickening. FFA: Multiple areas of leakage, a swelling of the optic disc.	Variant 1 Variant 2 Variant 3	No	CSF pleocytosis;	IV methylprednisolone, PO prednisolone, and topical steroids	4	NR	Resolution
Takeuchi 2023 [60]	71 F	Melanoma	Pembrolizumab	NR	NR	NR	NR	No	No	NR	Sub-tenon steroids, PO prednisolone	NR	NR	Resolution
Takeuchi 2023 [60]	69 F	RCC	Nivolumab	NR	NR	NR	NR	No	No	NR	Sub-tenon, topical steroids	NR	NR	Resolution
Takeuchi 2023 [60]	60 F	SCLC	durvalumab	NR	NR	NR	NR	No	No	NR	Sub-tenon, topical steroids	NR	NR	recurrence
Takeuchi 2023 [60]	54 F	Gastric and renal cancer	Nivolumab	NR	NR	NR	NR	No	No	NR	PO methylprednisolone, topical steroids	NR	Death	NR
Takeuchi 2023 [60]	81 M	Melanoma	Ipilimumab and Nivolumab	NR	NR	NR	NR	No	No		Sub-tenon, topical steroids	NR	NR	recurrence
Tamura 2018 [51]	61 M	NSCLC	Pembrolizumab	Ocular pain	NR	NR	OCT: Normal lining of the RPE. FFA: Granular leakage of fluorescein. Leakage of fluorescein from the optic disc is observed.	Variant 2 Variant 3	No	Hearing loss; CSF pleocytosis;	Corticosteroid, unspecified	NR	Death	Resolution
Tieger 2023 [52]	55 M	RCC	Nivolumab	Visual impairment and scotomas	Unremarkable	OU: Absence of vitritis, multifocal serous RD.	OCT: Choroidal thickening OU. FFA: Multiple areas of pinpoint leakage followed by pooling in the areas of serous detachments.	Variant 1 Variant 2	No	Vitiligo; Hearing loss;	PO prednisolone	0.1	NR	Resolution
Ushio 2021 [53]	58 M	Lung Adenocarcinoma	Nivolumab	Visual impairment	NR	NR	OCT: Serous RD, wavy RPE, thickening of choroid OU. ICGA: Patchy hypofluorescence of choroid. FFA: Hyperfluorescence of optic disc and granular hyperfluorescence on posterior pole.	Variant 1 Variant 2 Variant 3	Variant 1 Variant 2 Variant 3	Tinnitus; Hearing loss; Nausea/Vomiting;	Topical betamethasone, then IV and PO hydrocortisone after complications	NR	NR	Resolution
Wang 2024 [54]	69 M	Bladder Urothelial Carcinoma	Torpalimab	Ocular pain and vision loss	OU: Shallow AC, moderate inflammatory cells in AC, lens opacity, pigmentation on anterior capsule of lens.	OU: Posterior synechiae, optic disc swelling, peripheral serous RD.	OCT: Choroid thickened, bacillary layer, serous RD, fluid. FFA: Pinpoint hyperfluorescence. Punctate hyperfluorescence of optic disc OU.	Variant 1 Variant 2 Variant 3	No	NR	PO prednisone, dexamethasone sustained release implant OU	1	NR	Resolution
Witmer 2017 [55]	54 M	Melanoma	Ipilimumab	Blurred vision and floater	OU: 2 + granulomatous KP in the inferior cornea, AC 1 + cell.	OU: Trace vitritis, multiple white choroidal lesions in mid-periphery, a sunset glow fundus, trace optic disc edema.	OCT: Vitritis OU, trace subretinal fluid OS, optic disc edema. FFA: Staining of optic disc OU with late leakage.	Variant 1 Variant 3	Variant 1 Variant 2 Variant 3	Poliosis; Vertigo; Fever; Meningismus; Nausea/Vomiting;	PO dexamethasone	1.5	NR	Resolution
Wong 2012 [56]	43 F	Melanoma	Ipilimumab	Blurred vision	OU: Shallow AC.	OU: Trace vitreous cells and serous RD with associated choroiditis.	OCT: Intraretinal and subretinal fluid. FFA: Multiple areas of pinpoint leakage.	Variant 1 Variant 2	No	Poliosis; Vitiligo; Headache;	IV steroids and PO steroids	0.1	NR	Partial resolution
Yoshida 2020 [57]	63 M	Melanoma	Ipilimumab	Visual impairment	NR	OU: Numerous small, choroidal, depigmented atrophic lesions after uveitis resolution. At follow-up, sunset glow.	OCT: Uveitis. FFA: Hyperpermeability of the retinal blood vessels.	Variant 2	No	Poliosis; Vitiligo;	Sub-tenon triamcinolone and PO prednisone	NR	Complete remission	NR

AC = anterior chamber; ADA = adalimumab; F = female; FFA = fundus fluorescein angiography; ICGA = indocyanine green angiography; IV = intravenous; KP = keratic precipitates; M = male; MMF = mycophenolate mofetil; NR = not reported; NSCLC = non small-cell lung cancer; OCT = optical coherence tomography; OD = oculus dexter; OS = oculus sinister; OU = oculus uterque; OVD = ophthalmic viscosurgical device; PO = per os; RCC = renal cell carcinoma; RD = RD; RPE = retinal pigment epithelium; SCLC = small-cell lung cancer

### General clinical features of VKH-like uveitis

Overall, 42 patients (80.8%) had imaging or exams suggesting bilateral involvement [20–44, 46–48, 50, 52–56, 58, 59, 61], 3 (5.8%) suggested unilateral involvement [58, 62], and 7 (13.5%) did not report exam or test results. Twenty one cases (40.4%) reported data supporting panuveitis [20, 22–24, 26, 29, 35, 38, 40–44, 46, 48, 50, 54, 55, 58, 59, 61], 13 (25.0%) supported posterior uveitis [24, 25, 27, 28, 30, 33, 34, 52, 53, 56, 59, 62], 6 (11.5%) supported anterior uveitis [21, 31, 32, 39, 47, 59], 1 (1.9%) had normal ocular exam and imaging [36], and 11 (21.2%) did not report exams and imaging [37, 49, 51, 57, 58, 60]. 

From the reported ocular and imaging findings, 31 patients (59.6%) met the acute initial-onset uveitis criteria, of which 27 (51.9%) met criteria for variant 1 [22, 24, 26–30, 34–36, 38, 40, 42–44, 46, 50, 52–56, 59, 61], 21 (40.4%) for variant 2 [25, 26, 28, 30, 34–36, 38, 40, 42–44, 46, 50–54, 56, 57, 61], and 15 (28.8%) for variant 3 [20, 22, 26, 28, 34, 35, 40, 42, 43, 46, 50, 51, 53–55]. Thirteen cases (0.25%) met the late phase criteria, of which 7 (13.5%), [35, 42–44, 46, 53, 55] 9 (17.3%), [28, 29, 35, 38, 43, 44, 46, 53, 55] and 13 cases (25%) [24, 28, 29, 35, 38, 40, 42–44, 46, 53, 55, 61] met criteria for variant 1, 2, and 3 respectively. All late phase patients also met criteria for acute initial-onset uveitis. Altogether, 31 cases reported ocular or imaging findings that met either the acute initial-onset or late phase criteria of VKH disease [20, 22, 24–30, 34–36, 38, 40, 42–44, 46, 50–57, 59, 61]. The prodromal phase of the disease was reported by 5 cases (9.6%) [28, 34, 38, 55, 56]. Chronic recurrent disease was described in 11 cases (21.1%) [20, 22, 26, 33, 39–42, 59, 61]. Of these, sunset glow fundi were described in 8 cases [25, 33, 35, 41, 42, 44, 55, 57], and other chorio-retinal changes were described in 5 [23, 43, 46, 47, 53]. Only three reports described ICI-induced VKH-like uveitis in the absence of both extraocular symptoms and clinical findings that fit with the VKH disease criteria [39, 58, 60]. 

According to the old VKH disease criteria, only 12 of 52 cases (23.1%) met the complete VKH uveitis diagnosis criteria [20, 22, 23, 25, 27, 30, 38, 46, 52, 55, 56, 62], 15 (28.8%) met the incomplete criteria [24, 26, 28, 30, 34, 37, 41, 43, 47, 50, 51, 53, 57, 58, 61], 13 (25%) met the probable criteria, and 12 (23.1%) did not meet the diagnosis criteria for failing to demonstrate bilateral involvement of the disease. Among the latter, ten failed to report imaging findings with equivocal fundus exams [21, 31, 32, 49, 59, 60] and two had inconclusive imaging findings, namely trace nerve staining with retinal pigment epithelium atrophy [33] and choroidal thickening alone [48]. 

Visual impairment emerged as a key aspect of VKH-like uveitis based on the above findings. Therefore, best corrected visual acuity (BCVA) values are summarized in Table 6 for the cases where they were reported. The mean BCVA was 0.48 ± 0.43 logMAR, and the mean IOP was 17.2 ± 11.3 mmHg.

**Table 6:** Best corrected visual acuity and intraocular pressure

	BCVA (logMAR)		IOP (mmHg)	
Author Year	OD	OS	OD	OS
Monferrer-Adsuara 2021	0.301	0.176	12	13
Bricout 2017	0	0	18	16
Chaudot 2022	0.176	0.398	NR	NR
Conrady 2017	1	1.301	NR	NR
Conrady 2017	CF	HM	H	H
Crosson 2015	0	0	11	11
de Vries 2022	0.204	0	10	10
Dolaghan 2019	NR	NR	NR	NR
Enomoto 2020	0.217	0	10	13
Fierz 2016	0.217	1.301	35	43
Huang 2023	0.301	0.544	N	N
Hwang 2022	0.699	0.301	N	N
Kikuchi 2020	1	0.398	NR	NR
Kilani 2023	CF	CF	16	16
Kim 2019	0.176	0.699	NR	NR
Pedroso 2024	1	1	NR	NR
Madoe 2023	0.498	0	NR	NR
Matsuo 2017	0.187	0.187	14	14
Minami 2021	0.301	1.699	13	13
Nagai 2023	0.699	0.398	NR	NR
Ng 2023	1	0.301	NR	NR
Obata 2019	0.146	0.398	8	11
Pole 2023	CF	0.301	N	N
Reid 2019	0.477	0.602	8	8
Suwa 2021	0.699	0.398	11	12
Tieger 2023	0	0.477	NR	NR
Ushio 2021	0.097	1.097	16	17
Wang 2024	1	1	49	48
Witmer 2017	0.097	0.301	21	19
Wong 2012	0.176	0.699	NR	NR
Qian 2024	1.301	0.301	NR	NR
Qian 2024	1	1	NR	NR
Qian 2024	0.097	0.699	NR	NR

** HM = hand motion, CF = counting fingers, H = high, N = normal, NR = not reported

### Ocular symptoms

When analysing the VKH symptoms themselves (Tables 7 and 8), 38 of 52 (73.1%) patients reported bilateral ocular symptoms at presentations [20–22, 24–39, 41–43, 46–59]. Four patients (7.7%) reported unilateral symptoms. Ocular symptoms experienced by patients were not reported in ten cases [59–62]. The most common complaint found in 19 (36.5%) patients was subjective complaint of blurred vision and vision loss [22, 24, 26, 28, 30, 36, 38, 39, 42, 46–48, 50, 52–54, 57] and, in 18 (34.6%), blurry vision [20, 21, 25, 27, 29, 31–36, 40, 43, 49, 55, 56, 58, 59]. Signs typical of anterior chamber (AC) inflammation was reported as eye redness in five cases [20, 22, 26, 58, 59], photophobia in four [24, 32, 33, 58], and ocular pain in only 3 (5.7%) [33, 51, 54]. Symptoms suggestive of posterior chamber inflammation were reported by six patients, including two who accused of scotoma in one [58] or both eyes [52], two of bilateral floaters [42, 55], one of xanthopsia [41], and one of temporal photopsia [44]. 

**Table 7:** Summary of VKH-like Uveitis Presentation

Variables	Frequency (%)
Ophthalmic Findings	
Vision loss, n (%)	20 (37.7)
Blurry vision, n (%)	18 (33.9)
Ocular pain, n (%)	3 (5.7)
Not reported or other, n (%)	13 (24.5)
Slit Lamp Findings	
Anterior chamber reaction, n (%)	26 (50.0)
Keratic precipitates, n (%)	8 (15.4)
Vitreous cells, n (%)	4 (7.7)
Unremarkable, n (%)	9 (17.3)
Not reported, n (%)	17 (32.7)
Fundoscopy Findings	
SRD, n (%)	20 (41.5)
Optic disc edema, n (%)	11 (22.6)
Choroid lesions or atrophy, n (%)	12 (23.1)
Sunset glow fundus, n (%)	8 (15.4)
Not reported (%)	15 (28.8)
Imaging Findings	
SRD, n (%)	27 (51.9)
Choroidal thickening, n (%)	17 (32.7)
Early punctate staining and late subretinal dye pooling on FFA, n (%)	20 (38.5)
Hyperfluorescence of the optic disc on FFA, n (%)	16 (30.8)
Window defects/moth-eaten fluorescence on FFA, n (%)	10 (19.2)
Not reported or unsupportive of VKH, n (%)	18 (34.6)

SRD = serous retinal detachment

**Table 8:** Summary of VKH Treatment & Outcomes

Variables	Frequency (%)
Treatment	
PO steroids, n (%)	39 (75.0)
Topical steroids, n (%)	29 (55.8)
IV steroids, n (%)	15 (28.8)
Ocular steroid injections, n (%)	7 (13.5)
Non-steroidal immunosuppressants, n (%)	5 (9.6)
Not reported, n (%)	1 (1.9)
General Neoplastic Outcome	
Complete remission, n (%)	12 (23.1)
Partial remission, n (%)	8 (15.4)
Recurrence or progression, n (%)	5 (9.6)
Death, n (%)	3 (5.8)
Not reported, n (%)	24 (46.2)
Ophthalmic Outcome	
Resolution, n (%)	30 (57.7)
Partial resolution, n (%)	13 (25.0)
Progression or recurrence, n (%)	4 (7.7)
Not reported, n (%)	5 (9.6)

N.B. Ocular steroid injection includes intravitreal, peribulbar, subconjunctival, and sub-tenon modes of administration

### Slit lamp examination

Slit lamp findings were available in 35 patients, of which one case had unilateral findings only (left AC and vitreous cells) [47]. The predominant finding was general bilateral AC reaction or cells in 26 patients (50.0%) [20–24, 26, 29, 31–33, 35, 38, 40, 42, 43, 46–48, 50, 54, 55, 58, 59, 61]. Of these, eight were described as having keratic precipitates (KP) (30.8%), [20, 32, 35, 38, 42, 43, 46, 55] of which seven had granulomatous KPs with a mutton-fat appearance (26.9%) [20, 32, 35, 38, 42, 46, 55]. Three cases described fibrin formation in the AC [21, 29, 50]. Four cases reported posterior synechiae [20, 21, 48, 59], of which one was unilateral [48]. One case described as “panuveitis” had bilateral ciliary body detachments [24]. One case had unspecified bilateral ‘VKH-like panuveitis’ [59]. Four cases reported the presence of bilateral vitreous cells [26, 33, 46, 48], of which two were described as “mild” [26, 46]. Unremarkable or slit lamp examinations with no signs of inflammatory response were described in 9 cases (Table 5) [24, 25, 28, 34, 36, 41, 44, 52, 56]. 

### Fundus examination

Overall, 15 (28.8%) cases did not specify fundoscopy findings [21, 23, 24, 32, 37, 49, 51, 53, 57, 59, 60], and four cases reported unilateral findings only, described as “VKH-like reactions” [58] and unilateral SRDs [44, 62]. Twenty cases (38.5%) reported bilateral SRD [22, 24, 26, 27, 30, 35, 38, 40–43, 46, 54, 56, 58, 59, 61], two cases reported unilateral SRD in the right eye [28, 44], and one reported unilateral SRD with central serous retinopathy in the left eye [62]. Choroidal folds or thickening without serous retinal detachment were described in four bilateral cases [28, 33, 34, 39] and one unilateral case [29]. Eleven cases (21.2%) described bilateral optic disc edema [26, 29, 31, 33–35, 40, 48, 50, 54, 55], and two cases described bilateral optic disc hyperemia without disc swelling [24, 42]. Most cases of optic disc edema occurred in the absence of serous retinal detachment (*n* = 7, 63.6%) [29, 31, 33, 34, 48, 50, 55]. Bilateral choroidal lesions or hypopigmentation were noted in 5 (9.6%) cases – all occurred in the absence of subretinal fluid or SRDs [23, 25, 29, 46, 55]. Other fundus findings included one case being described as showing bilateral “intense vitritis” in the presence of AC reaction and granulomatous KPs [20], one case as bilateral “VKH-like reaction” with recurrent anterior uveitis [58], and one as unspecified bilateral posterior uveitis with anterior/intermediate uveitis [61]. One case described a mild narrowing of the retinal arterial vessels and scarce vitreous cells as the only clinical findings, along with a normal slit lamp exam [36]. 

### Imaging findings

The slit lamp and fundoscopy findings were supported by additional imaging including optical coherence tomography (OCT), fundus fluorescein angiography (FFA), and indocyanine green angiography (ICG) for 35 of 52 included cases (Table 5). OCT was reported in 30 patients, of which the most notable findings were the presence of characteristic serous retinal detachment/subretinal fluids (*n* = 15, 28.8%), [24, 27, 28, 34, 35, 40–42, 54–56, 58, 62] choroidal folds (*n* = 10, 19.2%), [22, 28, 40, 43, 44, 46, 50, 53, 58, 61] or choroidal thickening (*n* = 16, 30.8%) [22, 28, 30, 34, 36, 39–43, 48, 50, 52–54, 58, 61]. Three cases reported the presence of all three signs [28, 40, 58]. Two cases reported normal OCT images [20, 51]. 

ICG findings were reported in 10 patients, of which 7 (13.5%) noted hypofluorescent dark dots [28, 29, 42–44, 53, 61] and 3 (5.8%) stromal vessel hyperfluorescence and leakage [26, 35, 36]. Additionally, one case reported fuzzy choroidal vascular details (*n* = 1) [36].

FFA results were available in 24 cases [20, 22, 25, 26, 28, 33–36, 38, 40, 42–44, 46, 50–57, 61]. Of these, leakage of fluorescein was reported in 22 cases, either in the fundus (*n* = 9), [38, 44, 50, 52, 54, 56, 57, 61] optic discs (*n* = 4), [20, 22, 33, 55] or both (*n* = 11) [26, 28, 34–36, 40, 42, 43, 46, 51, 53]. Of these, pinpoint peripapillary hyperfluorescence were reported in 5 cases [46, 52–54, 56]. Starry-sky late hyperfluorescence was reported in 8 [25, 26, 36, 38, 44, 46, 53, 54, 61]. Chronic pigmentary changes with markedly pigmented areas adjoining hypopigmented zones (“moth-eaten appearances”) were reported in 9 cases [28, 29, 35, 42–44, 46, 53, 61]. Overall, imaging findings that were supportive of VKH-like uveitis diagnosis were reported in all but one case of 34 [62]. 

### Genotyping findings

Genotype findings were reported in 18 of 52 patients [21, 22, 25, 28, 30, 33, 35, 42, 43, 46, 50, 57, 60, 61]. Of these, 4 (7.7%) reported the presence of HLA-DR4 [42, 43, 50, 57], 10 (19.2%) the presence of HLA-DRB1*04, [21, 28, 30, 35, 60, 61 and 2 (3.8%) reported both [22, 61]. Of those reporting HLA-DRB1*04, the 04:05 variant was found in 9 out of 11 (81.8%) cases [28, 30, 35, 60, 61]. The other cases reported a heterozygous patient with the variants 04:10 and 04:06 [21], and another was unspecified [22]. 

### Extraocular symptoms

Over the course of illness, 33 (63.5%) patients presented extraocular symptoms associated with VKH, while 19 did not report any. Of extraocular manifestations, neurological and/or auditory symptoms were the most common, with 25 (48.1%) patients reporting at least one finding [20, 24–28, 30–32, 34, 38, 43, 46, 48, 49, 51–53, 55, 56, 62]. Of those, hearing loss was the most common, with 14 cases (26.9%), [20, 24, 27, 30–32, 38, 43, 48, 49, 51–53] followed by headaches (*n* = 8, 15.4%), [20, 25, 26, 30, 34, 38, 46, 56]. CSF pleocytosis (*n* = 6, 11.5%), [22, 23, 38, 43, 50, 51] vertigo (*n* = 5, 9.6%), [20, 31, 32, 49, 55] tinnitus (*n* = 4, 7.7%), [25, 28, 53, 62] meningismus (*n* = 2, 3.8%), [28, 55] nausea/vomiting (*n* = 2, 3.8%), [53, 55] and fever (*n* = 1, 2.0%) [55].

Twenty three cases (44.2%) reported at least one integumentary finding [20–23, 25, 27, 30–33, 37, 38, 41, 46–49, 52, 55–59, 61, 62]. Of those, fifteen cases presented with vitiligo (28.8%), [20–23, 25, 30, 33, 37, 52, 56–59, 61, 62] 15 with poliosis (28.8%), [32, 41, 46–48, 55–59, 61] and 3 with alopecia (5.8%) [32, 33, 38]. No cases presented with all three integumentary findings. Fourteen patients (26.9%) presented with both integumentary and neurological/auditory symptoms of VKH [20, 22, 23, 25, 27, 30, 32, 38, 46, 48, 52, 55, 56, 62]. 

Ten cases (19.2%) reported extraocular IRAEs over the course of ICI use. Namely, two cases reported adrenocortical insufficiency [20, 53]. One case reported erythema exudative multiforme [30]. One case reported ataxia [31]. One case reported type I diabetes mellitus [43]. One case reported transaminitis and hepatitis [52]. One had severe pneumonitis [59]. One had rash, myalgia, diarrhea, and transaminitis [58]. One developed interstitial lung disease and thyroid dysfunction [60]. Finally, one had duodenitis [62]. Overall, concomitant endocrine IRAEs were the most common (*n* = 4, 7.7%), followed by abdominal (*n* = 3, 5.8%), integumentary (*n* = 3, 5.8%), pulmonary (*n* = 2, 3.8%), and neurological/auditory (*n* = 1, 1.9%) extraocular IRAEs.

### Treatments

**Fig. 2:**
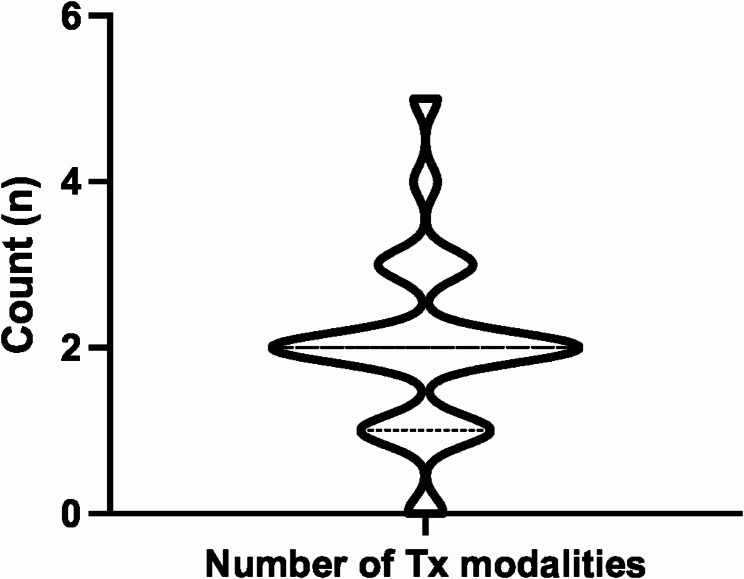
Violin plot showing the distribution and number of treatment modalities used to treat VKH-like uveitis

**Fig. 3:**
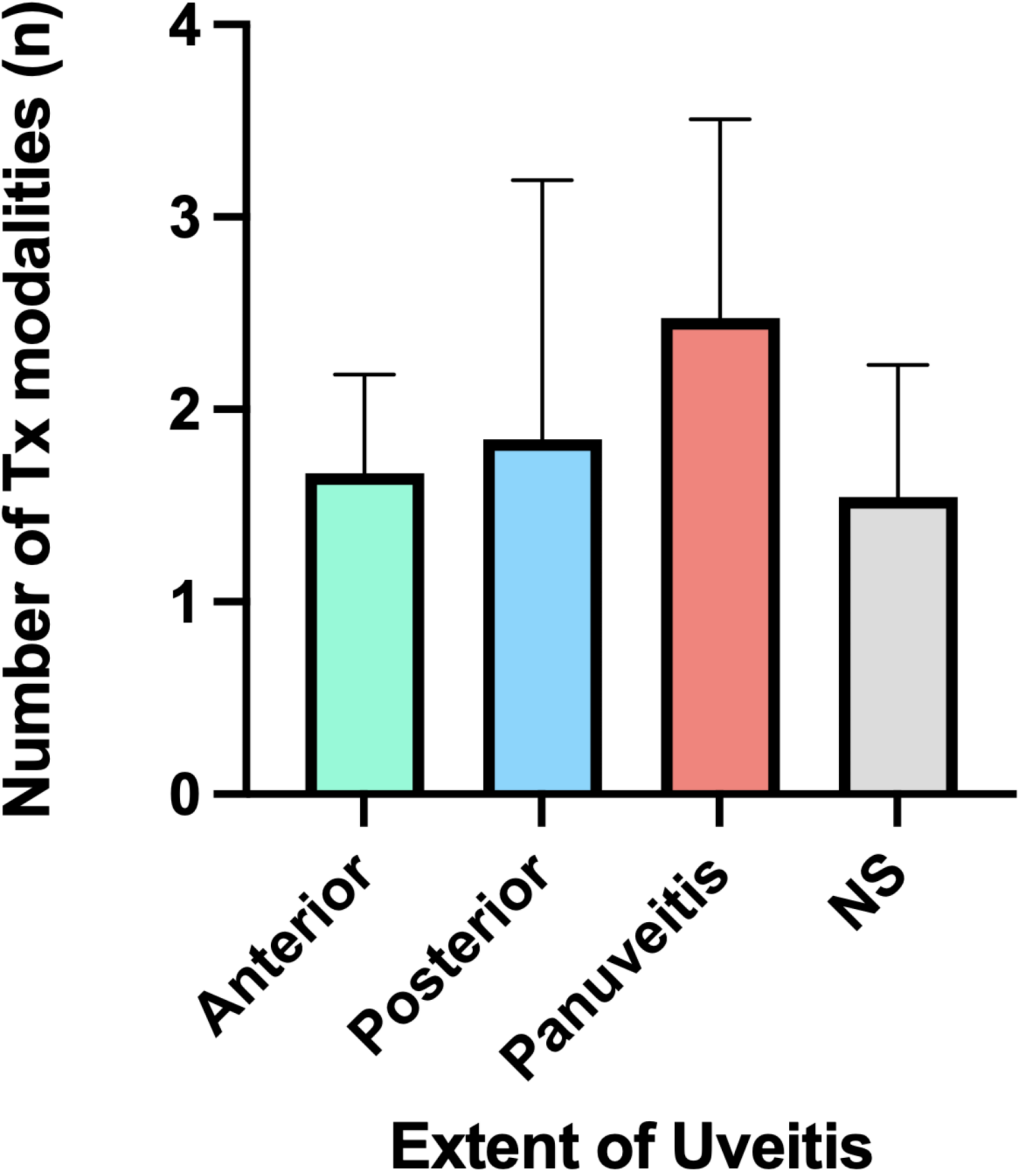
Number of treatment modalities used by authors according to the extent of VKH-like uveitis (mean ± SD)

For the patients included in our review, treatment information was reported in all but one report [62]. Only 5.8% (*n* = 3) and 3.8% (*n* = 2) of cases used biologics and antimetabolite immunosuppressants [32, 33, 59, 61]. All but one case were treated with steroids (98.1%), with 75.0% (*n* = 39) using oral steroids, [20, 23, 24, 26, 27, 28, 29, 30, 31, 32, 33, 34, 35, 36, 37, 38, 40, 41, 42, 43, 44, 48, 49, 50, 52–61] 55.8% (*n* = 29) using topical steroids, [20, 21, 22, 23, 24, 26, 27, 28, 29, 33, 35, 40, 41, 42, 43, 46, 49, 50, 53, 58, 59, 61] 28.8% (*n* = 15) using IV steroids, [23, 24, 30, 31, 34, 35, 36, 38, 42, 47, 48, 50, 53, 56] and 15.4% (*n* = 8) using sub-tenon steroids [33, 35, 42, 57, 60]. One patient received intravitreal corticosteroid [39], one received peribulbar triamcinolone, pars plana vitrectomy, and repeated injections of ophthalmic viscoelastic device and cross-linked hyaluronate into the anterior chamber [48], and one received corticosteroids with unspecified mode of administration [51]. Few patients (*n* = 4, 7.7%) described the use of mydriatrics [20, 21, 43, 46], Only one patient’s disease resolved with observation alone [25]. Most received combination treatments with only 8 cases reporting taking oral steroids alone [24, 37, 44, 52, 54, 55, 58], 2 cases undergoing IV steroids alone [30, 47], and 3 on topical steroid alone [21, 46, 58]. The distribution and number of treatment modalities reported by authors are illustrated in Fig. 2. The number of treatments was not significantly different between different diagnostic classifications of VKH but tended upward as fewer diagnostic criteria were met or reported by authors (Fig. 3A). Similarly, the mean number of treatment types tended upward in patients with signs of panuveitis, but this was not statistically significant (Fig. 3B).

Seventeen cases reported the timing of between treatment initiation and the time visual symptoms were first noted. Of those, 15 reported initiating some form of treatment in the first 4 weeks (88.2%), [24, 34–36, 41–43, 46, 48–50, 52, 54–56] while 13 initiated within the first 2 weeks (76.5%) [24, 34, 35, 41–43, 46, 48, 49, 52, 54–56]. None introduced immunosuppressants within 2–4 weeks.

**Fig. 4:**
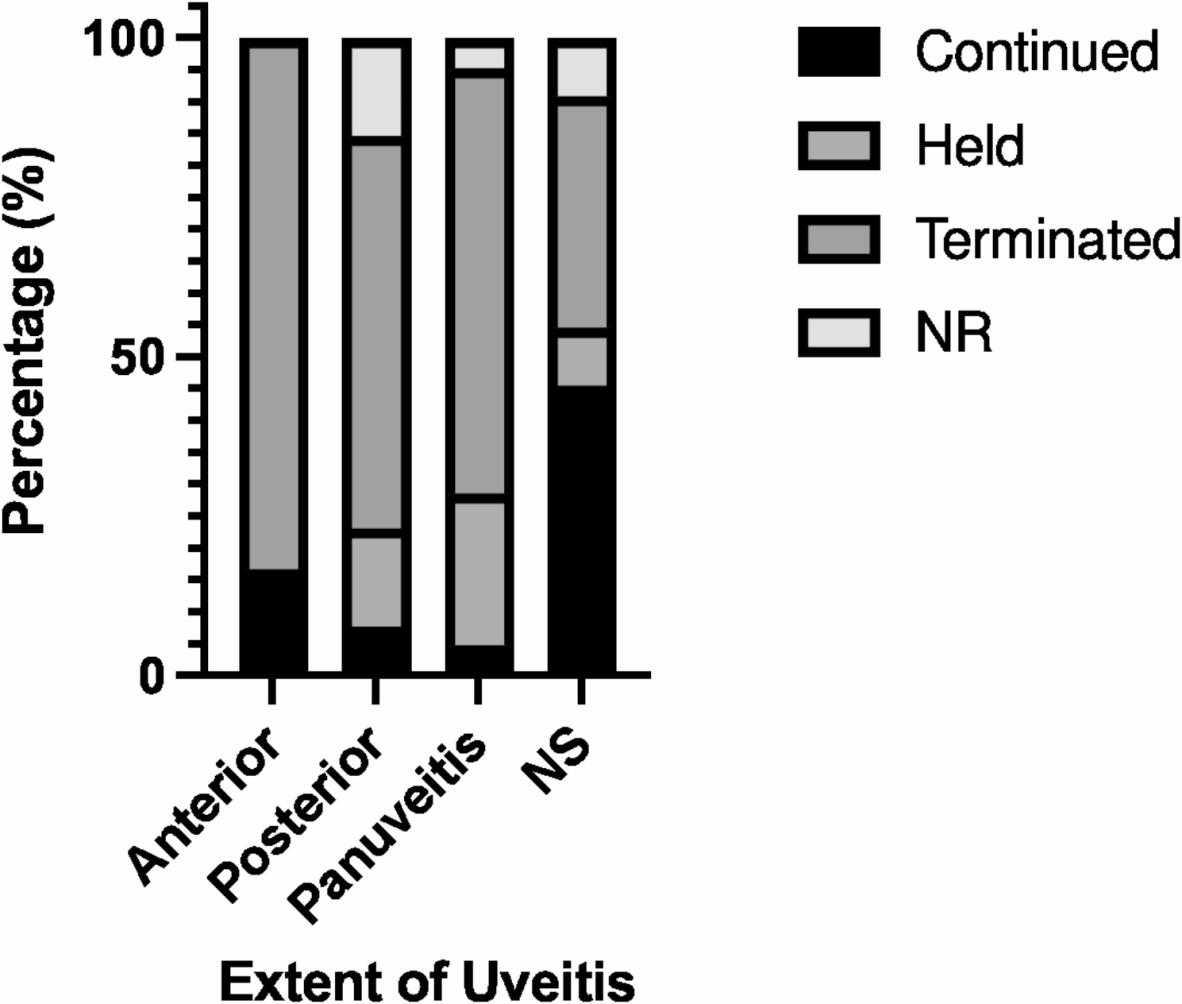
ICI treatment course of patients with ocular exams suggestive of anterior uveitis, posterior uveitis, and panuveitis. NS = not specified

ICI course was reported for 48 of 52 patients. Of these, more than half reported termination of ICI (*n* = 31, 59.6%) [20, 24, 26, 28, 29, 31–35, 39, 40, 43, 44, 46, 47, 50–56, 58–60]. Nine cases (17.3%) reported suspension for a median duration of 2.5 months (range = 1.75–12 months) [22, 30, 36, 41, 42, 48, 49, 61]. Finally, ICI was continued for 8 cases (15.4%) [21, 23, 57, 58, 60, 62]. Patients whose ocular exams were suggestive of anterior uveitis tended to have their ICI treatment continued, while those with suggestive posterior uveitis and panuveitis tended to suspend or terminate ICI courses though this was not statistically significant (Fig. 4).

### Visual and neoplastic prognosis

In terms of ophthalmic outcomes, 57.7% (*n* = 30) experienced complete resolution of VKH-like uveitis signs and symptoms, [20, 22, 23, 25, 28, 31, 34, 35, 37, 40, 43, 46, 47, 49, 51, 52, 53, 54, 55, 56, 57, 58, 60] 25.0% (*n* = 13) experienced partial resolution at the time of writing [27, 29, 30, 32, 36, 38, 39, 42, 44, 48, 50, 61], and only 7.7% (*n* = 4) experienced recurrences despite treatment [26, 33, 59]. Visual outcomes were not reported in 9.6% (*n* = 5) of cases [21, 24, 59, 62]. These findings are summarized in Table 5, and the detailed data in Table 8. Cancer prognosis after using ICI were favorable, with 23.1% reporting complete remission, and 15.4% stability of disease or partial remission. This is a sizable percentage, as 46.2% of patients’ outcomes were not reported. Among those reporting early treatment initiation (< 4 weeks), 66.7% (*n* = 10) experienced complete resolution of visual symptoms, 26.7% (*n* = 4) partial resolution, and 6.7% (*n* = 1) recurrence (Table 5).

**Fig. 5:**
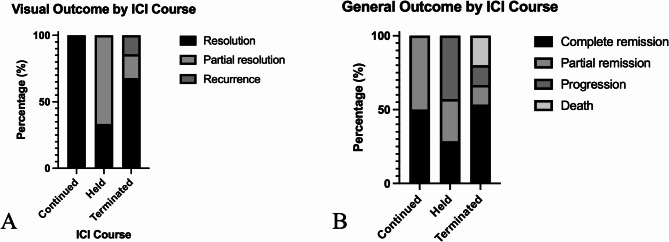
Visual (**A**) and general neoplastic (**B**) outcomes of VKH-like uveitis patients according to whether ICI was continued, held, or terminated

The visual outcomes of patients according to their ICI course is illustrated in Fig. 5A. Overall, among those reporting visual outcomes, 100% of VKH-like symptoms resolved by the end of the reported follow-up period for patients who continued ICI as prescribed. Among those who suspended ICI, 33.3% experienced full resolution, and the remaining experienced partial resolution of visual symptoms. Finally, among those who terminated ICI, 67.9% experienced full resolution, 17.9% experienced partial resolution, and 14.3% experience recurrence of ocular inflammation symptoms (*p* = ns).

The general cancer outcomes of patients according to their ICI course is illustrated in Fig. 5B. In sum, among those reporting general outcomes, 50% (*n* = 2) of patients who continued ICI as prescribed experienced complete remission [23, 62] and 50% (*n* = 2) experienced partial remission [21, 57]. Among those who suspended ICI, 28.6% (*n* = 2) experienced complete remission, [30, 41] 28.6% (*n* = 2) experienced partial remission, [22, 36] and 42.9% (*n* = 3) experienced cancer progression or recurrence [42, 48, 49]. Finally, among those who terminated ICI, 53.3% (*n* = 8) experienced complete remission, [26, 28, 31, 34, 40, 53, 55, 56] 13.3% (*n* = 2) experienced partial remission, [29, 32] 13.3% (*n* = 2) experienced progression, [33, 39] and 20% (*n* = 3) passed away (*p* = ns) [35, 59, 60].

**Fig. 6:**
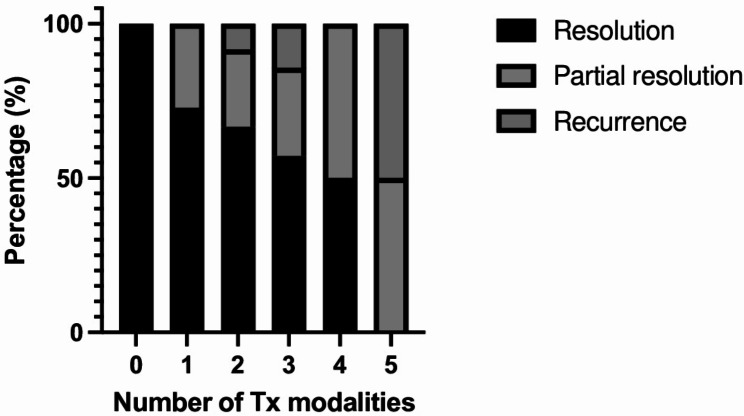
Visual outcomes according to the number of treatment modalities prescribed

Among those reported, better visual outcome seems to be associated with lower numbers of treatment modalities, but the severity of ocular symptoms is likely a confounder (Fig. 6). More severe VKH-like uveitis is likely to require more types of treatment and result in worse prognosis. Patients undergoing treatment with 1 or combinations of 2, 3, 4, and 5 types of treatment modalities experienced resolution of visual outcomes in 72.7%, 66.7%, 57.1%, 50.0%, and 0.0% of cases respectively. Partial resolution of visual signs and symptoms occurred in 27.3%, 25.0%, 28.6%, 50.0%, and 50.0% of cases respectively. Finally, recurrences occurred in 8.3% of patients undergoing combinations of two treatments, 14.3% for combinations of three, and 50.0% for combinations of five types of treatment.

### Quality of studies

**Fig. 7:**
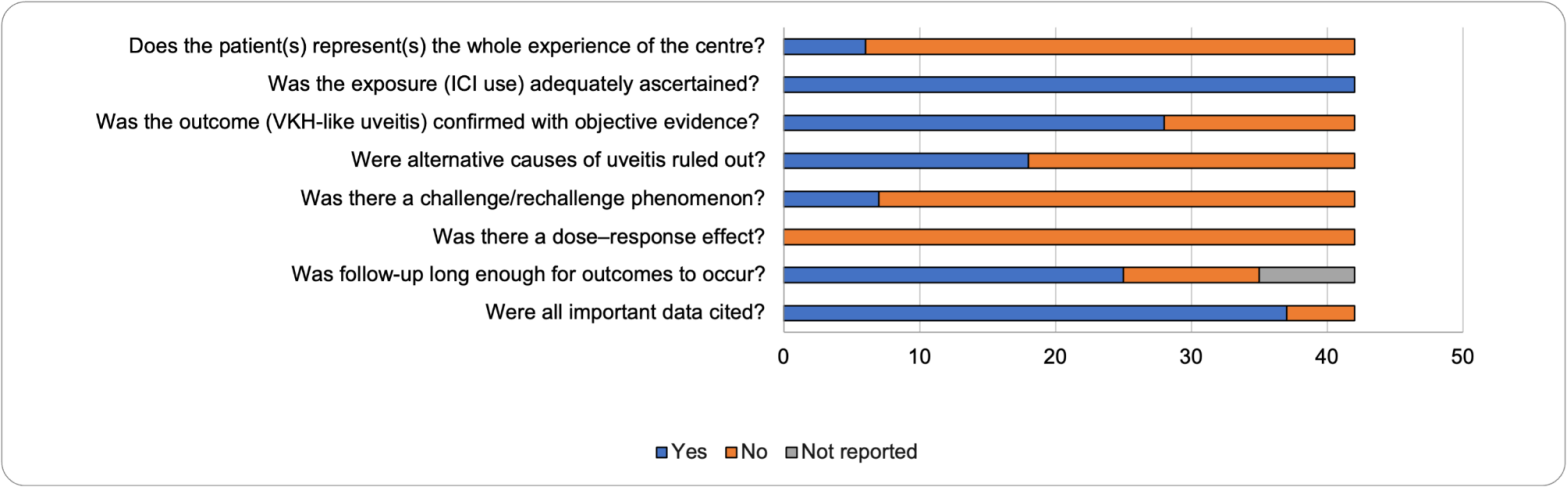
RoB assessment of the included articles

Finally, to analyse if bias was a major factor in these studies, we conducted a Risk of Bias (RoB) analysis, summarized in Fig. 7. Most of these reports were not complete as the experience of the whole centre, but all have adequately ascertained the exposure of immune checkpoint inhibitors prior to VKH symptoms. The literature did not extensively present processes considered when making the diagnosis of VKH, including what findings could be consistent with alternative causes of intraocular inflammation. Furthermore, only 31 (59.6%) patients fulfilled the acute initial-onset or chronic VKH disease criteria. However, evidence of the VKH was well supported overall, and follow-up periods were long enough for outcomes to occur after treatment. A vast majority of these papers also cited all important data and previous studies to support their conclusions. Overall, 95.2% (*n* = 40) were rated to have medium risk of bias, and only one article was deemed to have low risk of bias [22] and one to have high risk of bias [58]. Due to the case report nature of included articles, the certainty of evidence was rated as low.

## Discussion

Although ICI associated VKH-like uveitis is rare, it may alter treatment decision in cancer patients. Our search yielded 41 studies reporting on patient-specific outcomes. Our data showed that VKH-like uveitis associated with ICI is higher in patients with melanoma (36 patients out of 52 patients) and using PD-1 therapy. This supports the hypothesis that an excessive T-cell response against melanocyte antigens may underlie VKH-like uveitis pathogenesis [59]. In melanoma, melanocytes become cancerous by overly expressing PD-1 ligand, allowing them to evade the immune system and inhibit T-cell activity. PD-L1 plays a critical role in the maintenance of immune tolerance, T-cell activation and prevention of auto-immune reaction. When an ICI such as PD-1 inhibitor is used, it binds to and inhibits PD-L1 expression on tumor cells [60]. However, cross-reactivity may occur with melanocytes present in the choroid, leading to destruction of healthy retinal tissue [59]. By inhibiting the regulatory mechanisms of tolerance by PD-L1 and PD-1 on T cells, the use of ICIs results in overactivation of the immune system and loss of self-tolerance, resulting in VKH disease-like uveitis in the eye [61]. This immune overactivation can also affect other areas of the body that contain melanocytes such as the skin, hearing and central nervous system [59]. Therefore, presence of VKH-like uveitis in the context of ICI may correlate with positive therapy response and prognosis. In our review, about 26% of included patients had complete or partial tumoral response to ICI by the end of follow-up. Four studies report on the incidence of VKH-like uveitis, of which two articles examined ICI-induced uveitis cases signalled to adverse event databases and reported an incidence nearing 3.85% among all cases of ICI-induced uveitis – averaging an incidence of less than 1 in 2000 among all ICI IRAEs [62, 63]. On the other hand, Khanafer et al. report an incidence of 1.1% among 90 malignant melanoma patients treated at a French referral hospital, supporting a strong association between melanoma and ICI-induced VKH-like uveitis in a real-world setting [64]. 

Our study found that only 3 patients (5.8%) out of 52 were successfully treated with topical eye drops alone, and only 4 patients received non-steroidal immunosuppressants. Overall, there was a high burden of steroids for ICI-induced VKH-like uveitis – most cases reported some form of oral or intravenous steroids, and some reported sub-tenon steroid injections. Our result suggests that the majority of ICI-related uveitis reported as VKH-like was not treated within the window of therapeutic opportunity or using non-steroidal immunosuppression, the appropriate therapy for initial-onset VKH uveitis [13]. Indeed, numerous studies on VKH disease have shown that steroid monotherapy is not effective at preventing chronic disease, even if administered early on in the uveitis course, and that the addition of agents such as azathioprine, mycophenolate mofetil, cyclosporine, or anti-tumor necrosis factor alpha may play a pivotal role [11, 66–71]. 

We report a mean duration of ocular symptoms of 16.7 ± 18.6 weeks, meeting the 6-weeks criteria for chronic uveitis, although the variation is large (1–72 weeks). This is shorter than the duration of uveitis reported by Durrani et al. of 21 months in a cohort of consecutive eyes seen at an uveitis clinic, although no sub-group analyses were carried out to isolate for secondary or non-autoimmune uveitis [67]. Furthermore, it is often difficult to pinpoint with certainty the duration of uveitis because it may manifest without any ocular symptoms, and the duration of ocular symptoms could have been lengthened in some patients because of delayed diagnosis, inadequate steroids dose, or delay in suspending ICI as alternate causes of uveitis are ruled out. In our RoB analysis, we found that only 31 cases (59.6%) provided sufficient clinical details to meet diagnostic criteria for initial-onset or chronic uveitis associated with VKH disease [5]. Although a big proportion of cases reported as VKH-like uveitis failed to meet the diagnosis of “VKH uveitis”, almost all of those same cases reported extraocular symptoms traditionally associated with VKH disease. Additionally, only 1 article described a medical history of Type I Diabetes and one described the patient as a smoker, suggesting that VKH-like uveitis, unlike VKH disease, may be a distinct autoimmune entity occurring as a response to ICI therapy with a similar clinical presentation but unique disease course. VKH-like uveitis remains underrecognized in ICI therapy. While the above referred to the diagnostic criteria of VKH disease, a guideline specific to “VKH-like uveitis” has not been established and may provide clearer guidelines to future reporting.

The present study has various strengths. Our review followed PRISMA guideline for systematic review, and a librarian approved our search strategy. All articles that referenced VKH-like uveitis and ICI use were screened and extracted in duplicate by two reviewers to ensure accuracy and a systematic process. All included cases then underwent a standardized quality assessment by two individuals to ensure that the results were reliable and that the risk of bias was adequately assessed. Furthermore, a wide range of studies were included in this review including observational studies with patient-specific data and case reports. Thus, we adopted a modified RoB assessment tool adapted to studies reporting on individual outcomes, which is more suitable and specific to our aim. This allowed a more accurate assessment of the quality of included studies. Finally, detailed clinical information regarding treatment regimens and outcomes were evaluated, which contributes to a better understanding of the diagnosis and management of VKH-like uveitis. This is the first study to comprehensively evaluate reported cases of VKH-like uveitis in the setting of ICI.

Several limitations were identified. A significant proportion of the articles were case reports, which were based on single or few cases and reported in a non-systematic manner. This may lead to selection and outcome reporting bias, where more “interesting” cases are selectively reported. In fact, approximately one third of included studies were assessed as having a high RoB. Their anecdotal nature means we could not comment on risk factors of ICI-induced VKH-like uveitis. Additionally, our inclusion criteria required authors to conclude ICI-induced VKH-like uveitis as being the most likely diagnosis. Therefore, we could have excluded articles reporting on ICI-associated posterior uveitis that met VKH criteria but were not diagnosed as such by the authors. Finally, the reporting of outcomes were variable among included studies. For instance, few reported ICI dosage (*n* = 17) and duration (*n* = 20), leading to possible outcome reporting bias. Several larger observational studies reporting on VKH-like uveitis in ICI users were excluded because they did not report patient-specific outcomes [63, 64, 68]. However, we extracted valuable incidence information from these reports, contributing to our understanding of its epidemiology.

## Conclusion

This systematic review highlights the rare but significant occurrence of VKH-like uveitis as an immune-related adverse event in patients undergoing ICI therapy, especially in those with melanoma treated predominantly with PD-1 inhibitors. Findings indicate that VKH-like uveitis presents variably in clinical and imaging characteristics, often necessitating high-dose corticosteroid treatment for resolution, with a majority of patients achieving favorable ocular and cancer-related outcomes. The study underscores the need for standardized diagnostic criteria specific to VKH-like uveitis, distinct from traditional VKH disease, to enhance reporting consistency and guide clinical management. Despite limitations related to study heterogeneity and a high proportion of case reports, our review provides critical insights into the presentation, treatment, and outcomes of this underrecognized condition in the context of ICI therapy.

## Electronic supplementary material

Below is the link to the electronic supplementary material.

Supplementary Material 1

## Data Availability

Data generated or analyzed during this study are provided in full within the published article and its supplementary materials.
