# Supplementary material for: Immune checkpoint inhibitor-associated Vogt-Koyanagi-Harada-like syndrome: A descriptive systematic review
Source: J Ophthalmic Inflamm Infect. 2025 May 12;15:44. doi: 10.1186/s12348-025-00484-8 (PMC12069190; doi:10.1186/s12348-025-00484-8)
Supplement: Supplementary file 1 — Supplementary Material 1 [file 12348_2025_484_MOESM1_ESM.docx]

**Supplementary Figure 1:** Detailed search strategy

**Medline – Ovid on 28/06/2024**

((harada OR uveomening* OR uveo-mening* OR vogt-koyanagi*) ADJ3 (disease* OR syndrome*) OR (VKH OR "Vogt-Koyanagi-Harada" OR Uveomeningoencephaliti* OR uveo-meningo-encephaliti* OR Panuveiti* OR Sugiura OR "sunset glow fundus")).ti,ab,kf. OR Uveomeningoencephalitic Syndrome/ OR Panuveitis/ AND (("immune checkpoint*" OR CTLA-4 OR Cytotoxic-T-Lymphocyte-Associated-Protein-4 OR PD-1 OR programmed-Cell-Death-Protein-1 OR PD-L1 OR Programmed-Death-Ligand-1 OR PD-1-PD-L1) ADJ3 (block* OR inhibitor*)).ti,ab,kf. OR (Nivolumab OR Opdivo OR Ipilimumab OR Yervoy OR Pembrolizumab OR keytruda OR lambrolizumab OR xtrudane OR Atezolizumab OR tecentriq OR tecntriq OR Avelumab OR bavencio OR Durvalumab OR imfinzi OR Cemiplimab*).ti,ab,kf. OR Immune Checkpoint Inhibitors/ OR Nivolumab/ OR Ipilimumab/

**Embase** **on 28/06/2024**

(((harada OR uveomening* OR 'uveo mening*' OR 'vogt koyanagi*') NEAR/3 (disease* OR syndrome*)):ti,ab,kw OR 'vogt koyanagi syndrome'/exp OR 'uveitis'/de OR vkh:ti,ab,kw OR 'Vogt-Koyanagi-Harada':ti,ab,kw OR uveomeningoencephaliti*:ti,ab,kw OR 'uveo meningo encephaliti*':ti,ab,kw OR panuveiti*:ti,ab,kw OR sugiura:ti,ab,kw OR 'sunset glow fundus':ti,ab,kw) AND ((('immune checkpoint*' OR 'ctla 4' OR 'cytotoxic t lymphocyte associated protein 4' OR 'pd 1' OR 'programmed cell death protein 1' OR 'pd l1' OR 'programmed death ligand 1' OR 'pd 1 pd l1') NEAR/3 (block* OR inhibitor*)):ti,ab,kw OR nivolumab:ti,ab,kw OR opdivo:ti,ab,kw OR ipilimumab:ti,ab,kw OR yervoy:ti,ab,kw OR pembrolizumab:ti,ab,kw OR keytruda:ti,ab,kw OR lambrolizumab:ti,ab,kw OR xtrudane:ti,ab,kw OR atezolizumab:ti,ab,kw OR tecentriq:ti,ab,kw OR tecntriq:ti,ab,kw OR avelumab:ti,ab,kw OR bavencio:ti,ab,kw OR durvalumab:ti,ab,kw OR imfinzi:ti,ab,kw OR cemiplimab*:ti,ab,kw OR 'immune checkpoint inhibitor'/de OR 'nivolumab'/de OR 'ipilimumab'/de OR 'pembrolizumab'/de OR 'atezolizumab'/de OR 'avelumab'/de OR 'durvalumab'/de OR 'cemiplimab'/de)

**Web of Science on 28/06/2024**

(TS=((('immune checkpoint*' OR 'ctla 4' OR 'cytotoxic t lymphocyte associated protein 4' OR 'pd 1' OR 'programmed cell death protein 1' OR 'pd l1' OR 'programmed death ligand 1' OR 'pd 1 pd l1') AND (block* OR inhibitor*)))) AND (TS=(harada OR uveomening* OR 'uveo mening*' OR 'vogt koyanagi*'):)

**CENTRAL on 28/06/2024**

(((harada OR uveomening* OR uveo-mening* OR vogt-koyanagi*) NEAR/3 (disease* OR syndrome*)):ti,ab,kw OR MeSH descriptor: [Uveitis] explode all trees OR MeSH descriptor: [Uveomeningoencephalitic Syndrome] explode all trees OR vkh:ti,ab,kw OR 'Vogt-Koyanagi-Harada':ti,ab,kw OR uveomeningoencephaliti*:ti,ab,kw OR 'uveo meningo encephaliti*':ti,ab,kw OR panuveiti*:ti,ab,kw OR sugiura:ti,ab,kw OR 'sunset glow fundus':ti,ab,kw) AND ((('immune checkpoint*' OR 'ctla 4' OR 'cytotoxic t lymphocyte associated protein 4' OR 'pd 1' OR 'programmed cell death protein 1' OR 'pd l1' OR 'programmed death ligand 1' OR 'pd 1 pd l1') NEAR/3 (block* OR inhibitor*)):ti,ab,kw OR nivolumab:ti,ab,kw OR opdivo:ti,ab,kw OR ipilimumab:ti,ab,kw OR yervoy:ti,ab,kw OR pembrolizumab:ti,ab,kw OR keytruda:ti,ab,kw OR lambrolizumab:ti,ab,kw OR xtrudane:ti,ab,kw OR atezolizumab:ti,ab,kw OR tecentriq:ti,ab,kw OR tecntriq:ti,ab,kw OR avelumab:ti,ab,kw OR bavencio:ti,ab,kw OR durvalumab:ti,ab,kw OR imfinzi:ti,ab,kw OR cemiplimab*:ti,ab,kw OR MeSH descriptor: [Immune Checkpoint Inhibitors] explode all trees OR MeSH descriptor: [Nivolumab] explode all trees OR MeSH descriptor: [Ipilimumab] explode all trees)
